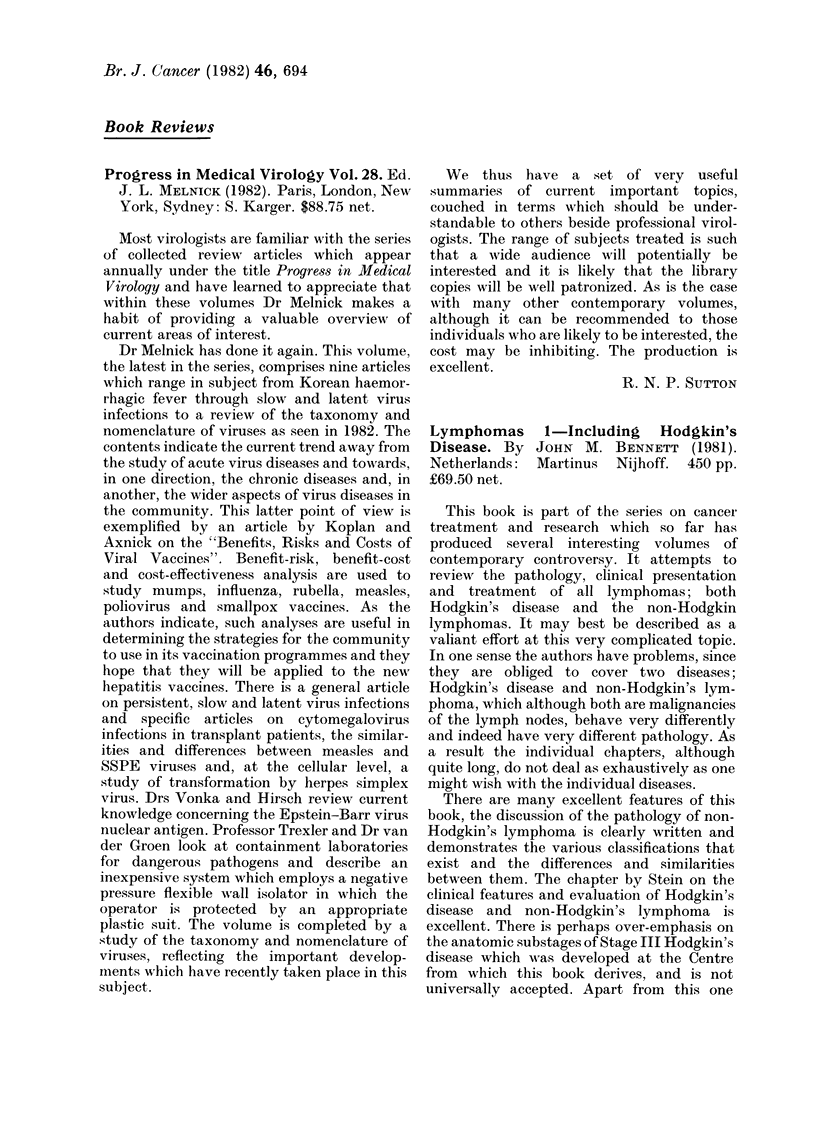# Progress in Medical Virology Vol. 28

**Published:** 1982-10

**Authors:** R. N. P. Sutton


*Br. J. Cancer* (1982) **46**, 694

### *Book Reviews*

**Progress in Medical Virology Vol. 28.** Ed. J. L. MELNICK (1982). Paris, London, New York, Sydney: S. Karger. $88.75 net.

Most virologists are familiar with the series of collected review articles which appear annually under the title *Progress in Medical Virology* and have learned to appreciate that within these volumes Dr Melnick makes a habit of providing a valuable overview of current areas of interest.

Dr Melnick has done it again. This volume, the latest in the series, comprises nine articles which range in subject from Korean haemorrhagic fever through slow and latent virus infections to a review of the taxonomy and nomenclature of viruses as seen in 1982. The contents indicate the current trend away from the study of acute virus diseases and towards, in one direction, the chronic diseases and, in another, the wider aspects of virus diseases in the community. This latter point of view is exemplified by an article by Koplan and Axnick on the "Benefits, Risks and Costs of Viral Vaccines". Benefit-risk, benefit-cost and cost-effectiveness analysis are used to study mumps, influenza, rubella, measles, poliovirus and smallpox vaccines. As the authors indicate, such analyses are useful in determining the strategies for the community to use in its vaccination programmes and they hope that they will be applied to the new hepatitis vaccines. There is a general article on persistent, slow and latent virus infections and specific articles on cytomegalovirus infections in transplant patients, the similarities and differences between measles and SSPE viruses and, at the cellular level, a study of transformation by herpes simplex virus. Drs Vonka and Hirsch review current knowledge concerning the Epstein–Barr virus nuclear antigen. Professor Trexler and Dr van der Groen look at containment laboratories for dangerous pathogens and describe an inexpensive system which employs a negative pressure flexible wall isolator in which the operator is protected by an appropriate plastic suit. The volume is completed by a study of the taxonomy and nomenclature of viruses, reflecting the important developments which have recently taken place in this subject.

We thus have a set of very useful summaries of current important topics, couched in terms which should be understandable to others beside professional virologists. The range of subjects treated is such that a wide audience will potentially be interested and it is likely that the library copies will be well patronized. As is the case with many other contemporary volumes, although it can be recommended to those individuals who are likely to be interested, the cost may be inhibiting. The production is excellent.

R. N. P. SUTTON